# Suboptimal Muscle Synergy Activation Patterns Generalize their Motor Function across Postures

**DOI:** 10.3389/fncom.2016.00007

**Published:** 2016-02-04

**Authors:** M. Hongchul Sohn, Lena H. Ting

**Affiliations:** ^1^George W. Woodruff School of Mechanical Engineering, Georgia Institute of TechnologyAtlanta, GA, USA; ^2^Wallace H. Coulter Department of Biomedical Engineering, Georgia Institute of Technology and Emory UniversityAtlanta, GA, USA

**Keywords:** motor modules, musculoskeletal model, postural response, isometric force, motor control

## Abstract

We used a musculoskeletal model to investigate the possible biomechanical and neural bases of using consistent muscle synergy patterns to produce functional motor outputs across different biomechanical conditions, which we define as generalizability. Experimental studies in cats demonstrate that the same muscle synergies are used during reactive postural responses at widely varying configurations, producing similarly-oriented endpoint force vectors with respect to the limb axis. However, whether generalizability across postures arises due to similar biomechanical properties or to neural selection of a particular muscle activation pattern has not been explicitly tested. Here, we used a detailed cat hindlimb model to explore the set of feasible muscle activation patterns that produce experimental synergy force vectors at a target posture, and tested their generalizability by applying them to different test postures. We used three methods to select candidate muscle activation patterns: (1) randomly-selected feasible muscle activation patterns, (2) optimal muscle activation patterns minimizing muscle effort at a given posture, and (3) generalizable muscle activation patterns that explicitly minimized deviations from experimentally-identified synergy force vectors across all postures. Generalizability was measured by the deviation between the simulated force direction of the candidate muscle activation pattern and the experimental synergy force vectors at the test postures. Force angle deviations were the greatest for the randomly selected feasible muscle activation patterns (e.g., >100°), intermediate for effort-wise optimal muscle activation patterns (e.g., ~20°), and smallest for generalizable muscle activation patterns (e.g., <5°). Generalizable muscle activation patterns were suboptimal in terms of effort, often exceeding 50% of the maximum possible effort (cf. ~5% in minimum-effort muscle activation patterns). The feasible muscle activation ranges of individual muscles associated with producing a specific synergy force vector was reduced by ~45% when generalizability requirements were imposed. Muscles recruited in the generalizable muscle activation patterns had less sensitive torque-producing characteristics to changes in postures. We conclude that generalization of function across postures does not arise from limb biomechanics or a single optimality criterion. Muscle synergies may reflect acquired motor solutions globally tuned for generalizability across biomechanical contexts, facilitating rapid motor adaptation.

## Introduction

It has been suggested that the nervous system may use a repertoire of fixed muscle patterns called muscle synergies, or motor modules, that can be flexibly combined to achieve functional motor goals. Muscle synergies have been shown to account for experimentally-observed variability in muscle activity across different motor behaviors in various species (Raasch and Zajac, 1999; Hart and Giszter, 2004; Ting and Macpherson, 2005; D'Avella et al., 2006; Chvatal et al., 2011; Roh et al., 2012). Each muscle synergy is hypothesized to produce a consistent biomechanical task (Giszter and Kargo, 2000; McKay and Ting, 2008). The level of recruitment of each muscle synergy during any given behavior is dependent on the spatiotemporal requirements of the task-level goal (Safavynia and Ting, 2013). For example, muscle synergies used for standing balance in cats and humans produced ground reaction force vectors that have distinct functions for controlling the center of mass (Ting and Macpherson, 2005; Chvatal et al., 2011). Muscle synergies used in human walking are associated with biomechanical sub-tasks such as body support, forward propulsion, or leg-swing (Neptune et al., 2009; Allen and Neptune, 2012; Lacquaniti et al., 2012).

Muscle synergies may represent robust motor solutions that are globally tuned for generalizability, which is defined as the ability to use same muscle activation pattern to produce functional motor outputs across different conditions. Generalizable motor solutions may simplify control and allow rapid adaptation to novel tasks (Wagner et al., 2007; Giszter and Hart, 2013; Tsianos et al., 2014; Minai, 2015; Ting et al., 2015). Experimental evidence suggests that the structure, or pattern of muscle synergies are robust across a variety of motor behaviors and biomechanical conditions. In cats, consistent muscle synergies and the ground reaction forces they produce (Ting and Macpherson, 2005) explained the reactive balance responses across a variety of postures (Torres-Oviedo et al., 2006). In humans, common muscle synergies are observed across variations in standing postures, reactive balance strategies (Torres-Oviedo and Ting, 2010; Chvatal et al., 2011), walking with altered loads (McGowan et al., 2010), reaching in various dynamic, and postural conditions (D'Avella et al., 2006), as well as during isometric force generation in multiple directions at different postures in human arm (Roh et al., 2012). Further evidence suggests that muscle synergies may even be shared across different motor tasks, such as feet-in-place and step responses during reactive balance (Chvatal et al., 2011), reactive balance and walking (Chvatal and Ting, 2013), forward and backward locomotion (Raasch and Zajac, 1999; Ting et al., 1999), and a range of different hindlimb motor tasks in frogs, such as jumping, swimming, kicking, and reflexive wiping (Hart and Giszter, 2004; Cheung et al., 2005, 2009; D'Avella and Bizzi, 2005; Roh et al., 2011).

However, the degree to which the generalization of muscle synergy functions across conditions results from properties of the biomechanical vs. neural control system has not been explicitly tested. Some studies suggest that biomechanical constraints may largely define the structure of muscle synergies (Kutch and Valero-Cuevas, 2012) such that generalization of function simply reflect similar biomechanical constraints across conditions. On the other hand, other studies suggest that muscle activation patterns reflected in muscle synergies arise from optimality criteria (Todorov, 2004; Steele et al., 2013; De Groote et al., 2014) that may specify similar solutions across conditions. However, experimental evidence suggests that there is no single muscle activation pattern used across individuals despite similar motor outputs (Torres-Oviedo et al., 2006; Torres-Oviedo and Ting, 2007; Clark et al., 2010; Chvatal et al., 2011; Frère and Hug, 2012). Our recent work suggests that muscle activity for performing a motor task in a *single condition* is largely unconstrained (Sohn et al., 2013; Simpson et al., 2015). Instead, a large number of “good-enough” solutions can be identified to perform any motor task (Raphael et al., 2010; Loeb, 2012), demonstrating our ability to take advantage of the highly redundant motor solution space. We showed that a wide range of activation levels for individual muscles is feasible for generating experimentally-observed endpoint forces in a static cat hindlimb model, suggesting that neither biomechanics nor single optimality considerations can fully explain experimentally-observed variations in muscle activation patterns and muscle synergies across subjects (Herzog and Leonard, 1991; Buchanan and Shreeve, 1996; Van Der Krogt et al., 2012; Sohn et al., 2013). It is likely that multiple criteria are required to explain neural principles for determining muscle synergy patterns (Ganesh et al., 2010).

Based on prior experimental findings demonstrating the generalizability of muscle synergy forces based on individual-specific muscle synergies across postures (Ting and Macpherson, 2005; Torres-Oviedo et al., 2006), we previously showed that the rotation of the muscle synergy force vectors can be predicted by applying a common simulated muscle synergy pattern across different postures (McKay and Ting, 2008). While the rotation of force vectors with respect to limb axis were largely similar across widely varying muscle synergy patterns that produced the same force, the differences in the generalizability of different feasible muscle synergy patterns was not explicitly tested. Further, we predicted that functional requirements for generalizability would narrow the range of possible muscle activation patterns for muscle synergies such that the selection of a particular muscle synergy could be influenced by its ability to satisfy biomechanical task constraints across a range of limb postures.

**Figure 1:**
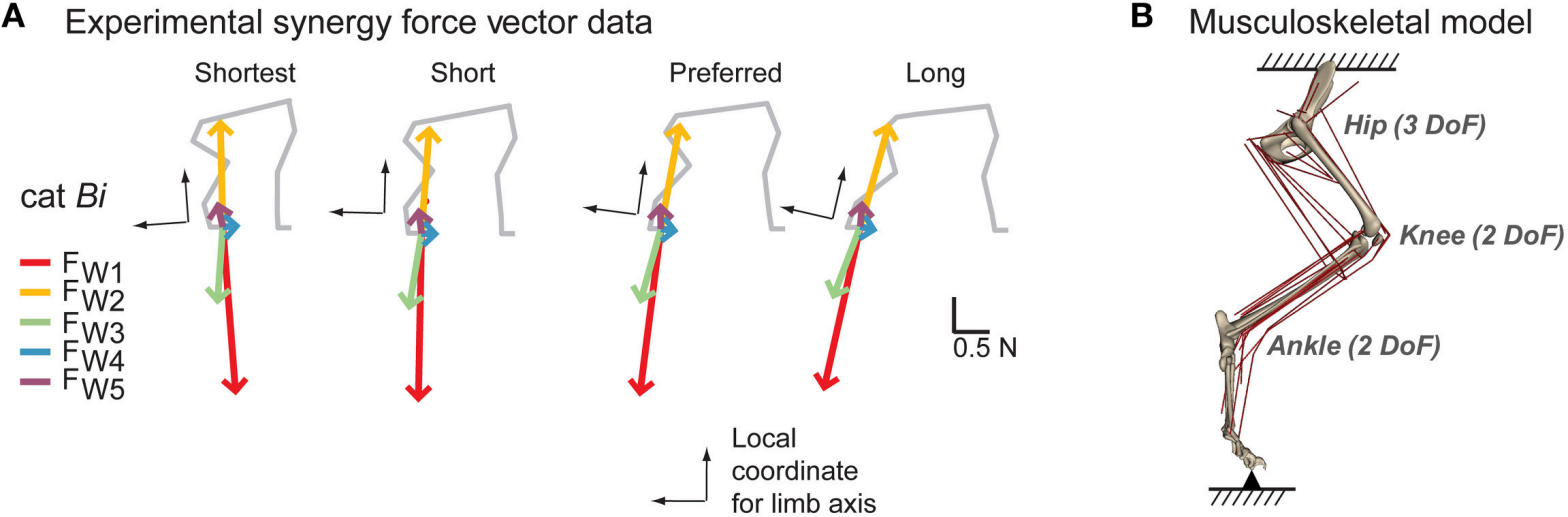
**Experimental data and model. (A)** Experimental synergy force vectors (F_W1~5_) in cat *Bi* from Torres-Oviedo et al., (2006). A common set of muscle synergies (W_1_~W_5_) explained reactive balance behavior in cats across different postural configuration and produced consistent endpoint force vectors with respect to limb orientation. The extensor synergy force vector (red) and the flexor synergy force vector (yellow) that were largest in magnitude and had the most consistent direction across cats were selected for the study. **(B)** Musculoskeletal model of the cat hindlimb (Burkholder and Nichols, 2004) with seven rotational degrees of freedom (3 at the hip, 2 each at the knee and ankle) and 31 muscles. In this static model, the pelvis was fixed to the ground and the endpoint (MTP joint) was connected to the ground via gimbal joint.

Here, we used a musculoskeletal model of the cat hindlimb to investigate the degree to which biomechanical properties of the limb vs. neural control mechanisms underlie the consistent muscle synergies observed across different limb postures. Rather than identifying muscle synergy patterns through numerical factorization analysis of a set of experimentally-measured or theoretically-derived muscle activation patterns (Tresch et al., 2006; Kutch and Valero-Cuevas, 2012; Steele et al., 2013, 2015; De Groote et al., 2014), we examined the set of biomechanically feasible muscle activation patterns to achieve a particular function (McKay and Ting, 2008, 2012; Sohn et al., 2013). Specifically, we explored the full set of candidate muscle activation patterns that could produce muscle synergy force vectors, i.e., experimentally-derived force vectors that were co-modulated with recruitment level of a particular muscle synergy during postural responses (Figure 1A). We use the term “muscle synergy” or “synergy force vector” when referring to experimental data used to motivate the study, and “muscle activation pattern” when referring to candidate vectors of muscle excitation that generate specific target endpoint forces in the model. This approach allowed us to test the principles by which the nervous system may select muscle activation patterns with generalizable function across different biomechanical conditions, which manifest as consistent muscle synergies associated with consistent synergy force vectors across multiple biomechanical conditions.

We tested two mutually exclusive hypotheses about how generalizability of muscle activation patterns across different biomechanical conditions may arise. First, we hypothesized that generalizability is a property of limb biomechanics such that all possible muscle synergy patterns generalize their function across postures. Second, we hypothesized that generalizability is a property of an optimal (e.g., minimum-effort) solution for each posture, such that optimal muscle synergies found across postures have similar patterns and functions. Finally, we hypothesized that generalizability reflects the selection of specific muscle synergy pattern that provides similar functions across postures over other muscle synergy patterns that do not generalize across postures. This hypothesis predicts that muscle synergy patterns that generalize their function across different biomechanical configurations are sub-optimal for producing a force at any single configuration. Further, generalizability constraint will reduce the range of possible muscle synergy patterns. To test these hypotheses, we examined muscle activation patterns, representing candidate coordination pattern for muscle synergies, that produced the experimentally-observed ground reaction force vectors across shortest, short, preferred and long stances. For each posture, we generated muscle activation patterns that produced the experimentally-observed force vector in that posture according to three different selection criteria: randomly selected feasible muscle activation patterns, a minimum-effort (min-E) muscle activation pattern, and a generalizable muscle activation pattern that minimizes deviations from the experimentally-observed force vectors across all postures. We found that only a few selected muscle activation patterns could generalize their output force direction across postures. Our results demonstrate that functional demands for generalization of muscle synergies across postures can affect the selection of muscle activation patterns, and does not arise from limb biomechanics or a single optimality criterion alone.

## Materials and methods

In summary, we used a detailed musculoskeletal model of the cat hindlimb to test the generalizability of theoretically possible muscle synergy patterns across postural configurations based on three different selection methods: (1) randomly selected feasible muscle activation patterns, (2) optimal muscle activation patterns minimizing muscle effort for a given posture (i.e., min-E muscle activation pattern), and (3) generalizable muscle activation patterns that explicitly minimized deviation from the experimentally-observed forces across all postures. For each posture, we generated muscle activation patterns that produced the experimentally-observed force vectors at a target posture. We then tested generalizability of these muscle activation patterns by applying them to three other stance postures and examining the resulting endpoint force vectors in the model. We assessed generalizability of each muscle activation pattern in terms of angular deviations in the resulting force vectors from the experimentally-observed force vectors at each test posture. We further tested whether feasible range of activation in individual muscles for producing the experimentally-observed force vector at preferred posture is reduced with a generalizability constraint.

### Experimental data and musculoskeletal model

#### Experimental synergy force vector

Experimentally-observed force vectors were taken from a previous study investigating reactive balance behavior in cats (Torres-Oviedo et al., 2006). Cats stood quietly at four different postures with varying fore-hindlimb stance distances: shortest, short, preferred and long (Macpherson, 1994). Across all stance postures, five muscle synergies (W_1_~W_5_) in each cat robustly explained active changes in muscle activity and hindlimb forces in response to multi-directional horizontal support-surface perturbations. Experimental synergy force vectors (${\overset{⇀}{F}}_{{\text{W}}_{\text{i}}}$) were found by extracting three-dimensional endpoint force vector components that were co-modulated with recruitment level of each muscle synergy. These synergy force vectors rotated with the limb axis across postures (Figure 1A). In this study we examined two synergy force vectors the extensor synergy force vector (${\overset{⇀}{F}}_{{\text{W}}_{1}}$; Figure 1A, red) and the flexor synergy force vector (${\overset{⇀}{F}}_{{\text{W}}_{2}}$; Figure 1A, yellow) because they had the largest magnitudes and the most consistent force directions across cats and postures, providing active loading and unloading of the limb during postural responses to perturbations (Torres-Oviedo et al., 2006).

#### Cat hindlimb model

We used a previously developed three-dimensional musculoskeletal model of the cat hindlimb (Figure 1B; Burkholder and Nichols, 2004). Details of this model are described elsewhere in both static (McKay et al., 2007; Sohn et al., 2013) and dynamic conditions (Bunderson et al., 2008, 2010). Briefly, the model included seven degrees-of-freedom at anatomical joints (3 at the hip, 2 at the knee, 2 at the ankle) and 31 hindlimb muscles (list and abbreviations in Table 1). The posture of the model was matched to kinematics of each cat at each of the stance configurations (McKay and Ting, 2008). Across postures, difference in joint angles were mostly in the sagittal plane (25~40° in hip, knee, and ankle extension/flexion angle). The pelvis was fixed to ground and the endpoint was defined at the metatarsal-phalangeal joint (MTP), which was connected to the ground via gimbal joint. At static equilibrium, the model defined a linear mapping between muscle activation and endpoint force:

(1) $$\begin{array}{r} RF_{AFL}\overset{⇀}{a}=J^{\text{T}}{\overset{⇀}{F}}_{End}, \end{array}$$

where **R** is the moment arm matrix (7 × 31), **F**_*AFL*_ is the diagonal matrix (31 × 31) of scaling factors for active muscle force generation, $\overset{⇀}{a}$ is the muscle activation vector (31 × 1), **J** is the geometric Jacobian (3 × 7), and ${\overset{⇀}{F}}_{End}$ is the endpoint force vector (3 × 1). Muscle moment arm (**R**), geometric Jacobian (**J**), and muscle parameters required to characterize active muscle force generation (**F**_*AFL*_), i.e., maximum isometric force and force-length relationship (Zajac, 1989), were acquired using *Neuromechanic*, a previously developed and freely-available software package (Bunderson et al., 2012).

**Table 1:** **Muscles included in the hindlimb model and abbreviations**.

**Name**	**Abbreviation**
*Adductor femoris*	ADF
*Adductor longus*	ADL
*Biceps femoris anterior*	BFA
*Biceps femoris posterior*	BFP
*Extensor digitorum longus*	EDL
*Flexor digitorum longus*	FDL
*Flexor hallicus longus*	FHL
*Gluteus maximus*	GMAX
*Gluteus medius*	GMED
*Gluteus minimus*	GMIN
*Gracilis*	GRAC
*Lateral gastrocnemius*	LG
*Medial gastrocnemius*	MG
*Peroneus brevis*	PB
*Pectineus*	PEC
*Peroneus longus*	PL
*Plantaris*	PLAN
*Iliopsoas*	PSOAS
*Peroneus tertius*	PT
*Pyriformis*	PYR
*Quadratus femoris*	QF
*Rectus femoris*	RF
*Sartorius*	SART
*Semimembranossus*	SM
*Soleus*	SOL
*Semitendinosus*	ST
*Tibialis anterior*	TA
*Tibialis posterior*	TP
*Vastus intermedius*	VI
*Vastus lateralis*	VL
*Vastus medialis*	VM

We used Hill-type muscle model with inelastic tendons, which are typically employed in static analyses of force production (Kuo and Zajac, 1993; Valero-Cuevas et al., 1998; Valero-Cuevas, 2000; McKay et al., 2007). Static properties of the musculo-tendon actuator were characterized by four muscle-specific parameters: peak isometric muscle force, optimal muscle-fiber length, optimal muscle fiber pennation angle, and tendon slack length, based on measured architectural properties of cat hindlimb muscles (Sacks and Roy, 1982; Roy et al., 1997). Muscle fiber lengths were set at 65% of optimal fiber length for all muscles at preferred posture so that muscles operated on the ascending slope on force-length relationship curve and were operating at ranges below optimal fiber length across all postures. However, because no compliance was assumed in tendons, at certain extreme postures such as long stance some muscles could operate at ranges on the force-length relationship curve where active force generation capability was very low.

The experimental synergy force vectors at each of the four stance postures in each of two *cats* (*Bi* and *Ru*) were used as the task constraint at each target posture. These forces vectors represent the active response of the cats following perturbation, measured as the change in the ground reaction force from the background level (Jacobs and Macpherson, 1996). Thus, pattern of muscle activation that produce the experimental synergy force vector at target posture can be regarded as candidate coordination pattern for muscle synergy, which also represents active change in muscle activity in response to perturbations (Ting and Macpherson, 2005). Muscle activation patterns that produced the experimental synergy force vector at target posture were found using the static linear model mapping muscle activation vector to net joint torque requirement for given task (Equation 1). The generalizability of muscle activation patterns were tested at all postures by simulating the resulting endpoint force vectors using *Neuromechanic*; the full dynamic model was forward integrated for 1 ms such that reaction forces satisfying the kinematic constraints were computed but before acceleration and thus other inertial and velocity-dependent forces were developed.

### Candidate muscle synergy patterns based on the three selection criteria

#### Feasible muscle activation patterns

In order to test whether generalizability is a property of all possible muscle activation patterns and thus a property of limb biomechanics, we tested whether muscle activation patterns randomly distributed within the feasible set of solutions at one posture can generalize their function to other postures. We first generated set of 200 feasible muscle activation patterns by finding the nearest solution (least-square projection) to each of the 200 random patterns (${\overset{⇀}{a}}^{0}$) that were uniformly distributed within the feasible muscle activation ranges (Sohn et al., 2013) for producing experimental synergy force vectors at each target posture (${\overset{⇀}{F}}_{{\text{W}}_{\text{i}}}^{\text{Posture}}$, where i stands for either the extensor or the flexor synergy). This optimization problem was solved using quadratic programming (*quadprog* in Matlab; MathWorks, Natick, MA):

(2) $$\begin{array}{l} \text{minimize }{\left(\overset{⇀}{a}-{\overset{⇀}{a}}^{0}\right)}^{\text{T}}\left(\overset{⇀}{a}-{\overset{⇀}{a}}^{0}\right),\\ \text{subject to }RF_{AFL}\overset{⇀}{a}=J^{\text{T}}{\overset{⇀}{F}}_{{\text{W}}_{\text{i}}}^{\text{Posture}},\;and\;{lb}_{m}\leq a_{m}\leq {ub}_{m}, \end{array}$$

where the lower (*lb*_*m*_) and upper bounds (*ub*_*m*_) of individual muscles were determined from the feasible muscle activation range. In order to ensure that the feasible muscle activation patterns span the full range of possible effort levels (sum-squared activation, i.e., ∑*a*^2^; Crowninshield and Brand, 1981) at each target posture, we included 50 additional muscle activation patterns that were generated by linearly scaling the difference in muscle space between the two solutions that had the minimum and maximum possible effort level.

These 250 feasible muscle activation patterns were tested at all postures and the resulting force angle deviations ($△\theta_{{\text{W}}_{\text{i}}}^{\text{Posture}}$, where i stands for either the extensor or the flexor synergy) were computed. Force angle deviations were computed using the angle defined by the inverse cosine of the normalized dot product between the experimental synergy force vector at each test posture (${\overset{⇀}{F}}_{{\text{W}}_{\text{i}}}^{\text{Posture}}$) and simulated endpoint force (${\overset{⇀}{F}}_{End}^{\text{Posture}}$) at each test posture:

(3) $$\begin{array}{r} △\theta_{{\text{W}}_{\text{i}}}^{\text{Posture}}={\text{cos}}^{-1}\left(\frac{{\overset{⇀}{F}}_{{\text{W}}_{\text{i}}}^{\text{Posture}}{\overset{⇀}{∙F}}_{End}^{\text{Posture}}}{∥{\overset{⇀}{F}}_{{\text{W}}_{\text{i}}}^{\text{Posture}}∥∥{\overset{⇀}{F}}_{End}^{\text{Posture}}∥}\right) \end{array}$$

#### Minimum-effort muscle activation pattern

To test whether a muscle activation pattern that is optimized in terms of effort at a single posture can be generalized across postural configurations, we assessed force angle deviations of an optimal muscle activation pattern at each posture across all of the postural configurations. The minimum-effort (min-E) muscle activation pattern was selected based on the criteria used most often in musculoskeletal modeling (Erdemir et al., 2007), minimizing sum of squared muscle activations (Equation 4; Crowninshield and Brand, 1981; Anderson and Pandy, 2001; Thelen et al., 2003). For each min-E muscle activation pattern, we used quadratic programming to identify a unique muscle activation vector that minimized sum of squared muscle activations while satisfying the experimental synergy force vector at target posture:

(4) $$\begin{array}{l} \text{ minimize }{\vec{a}}^{\text{T}}\vec{a}\;(\text{which is equivalent to}\sum a^{2}),\\ \text{subject to }RF_{AFL}\vec{a}=J^{\text{T}}{\vec{F}}_{{\text{W}}_{\text{i}}}^{\text{Posture}},\text{ and }lb_{m}\leq a_{m}\leq ub_{m}, \end{array}$$

Force angle deviations of the min-E muscle activation pattern at test postures were computed as in Equation 3 and were compared to those of muscle activation patterns selected from other criteria.

#### Generalizable muscle activation pattern

To determine whether a single muscle activation pattern can be found that can generalize its output force across different conditions, we explicitly searched for a single solution that best generalized experimental synergy force vectors across postures. We formulated a non-linear optimization problem to identify a unique muscle activation pattern that produced experimental synergy force vector at each posture while minimizing the deviation from experimentally-observed synergy force vector at all test postures:

(5) $$\begin{array}{l} \text{minimize }\left|△\theta_{{\text{W}}_{\text{i}}}^{\text{Shortest}}\right|+\left|△\theta_{{\text{W}}_{\text{i}}}^{\text{Short}}\right|+\left|△\theta_{{\text{W}}_{\text{i}}}^{\text{Preferred}}\right|+\left|△\theta_{{\text{W}}_{\text{i}}}^{\text{Long}}\right|,\\ \text{ subject to }RF_{AFL}\vec{a}=J^{\text{T}}{\vec{F}}_{{\text{W}}_{\text{i}}}^{\text{Posture}},\text{ and }lb_{m}\leq a_{m}\leq ub_{m} \end{array}$$

This non-linear optimization problem was solved using *fmincon* in Matlab. In order to ensure convergence to a global minimum solution, we performed the search using random initial conditions. Due to the computational intensity of forward simulations, we used 100 initial conditions in which activation levels of individual muscles were uniformly distributed across the feasible muscle activation range. After 100 searches converged to each minimum (Equation 5), we estimated the global minimum by selecting the solution in which the cost was the smallest across all initial conditions. Further analyses of the generalizable muscle activation pattern were based on this global minimum solution. Across conditions, 97 ± 6.8% of the searches converged to solutions with costs not larger than 0.01% of the global minimum. To examine possible redundancy in muscle space we further examined distribution of muscle activation across these near-minimal solutions.

### Analyses

#### Comparison of generalizability of muscle activation patterns across selection criteria

For each synergy force vector, we tested whether muscle activation patterns selected from different criteria differ in generalizability using pairwise Wilcoxon rank-sum tests, which are non-parametric tests for comparing between two groups (selection criteria). The dependent variables were force angle deviation at each test posture for each of selection criteria, i.e., (1) the mean force angle deviation for each set of the 250 feasible muscle activation patterns (*n* = 24; 2 cats × 4 target postures × 3 test postures), (2) the force angle deviation for min-E muscle activation patterns (*n* = 24), and (3) the force angle deviations for generalizable muscle activation patterns (*n* = 24). Significance was evaluated at α = 0.05, adjusted with a Bonferroni correction for multiple comparisons, i.e., α = 0.017 (=0.05/3). We also compared force angle deviations after removing outliers. We identified and removed the outliers in each group using interquartile range with Tukey's method (Tukey, 1977). We then conducted the same pairwise Wilcoxon rank-sum test with significance evaluated at α=0.017 with Bonferroni adjustment.

#### Effort level of the generalizable muscle activation patterns

We evaluated effort level of the generalizable muscle activation pattern and compared it to the min-E muscle activation pattern at each posture. The effort level of each muscle activation pattern was normalized to that of the maximum-effort possible at each posture, which was found using optimization similar to Equation 4 but with a cost function that maximized effort.

#### Effect of torque requirement on similarity in muscle activation patterns across postures

We tested whether different torque requirement across postures affected the spatial activation pattern of min-E or generalizable muscle activation pattern. We examined spatial similarity of muscle activation patterns across postures selected either from min-E criteria or generalizability criteria. In particular, we computed the similarity in terms of the cosine of the angle between 31-dimensional vectors of muscle activation patterns in all possible combinations:

(6) $$\begin{array}{r} {\left[\cos\theta \;\right]}_{{\overset{⇀}{a}}^{{\text{Posture}}_{i}},{\overset{⇀}{a}}^{{\text{Posture}}_{j}}}=\frac{{\overset{⇀}{a}}^{{\text{Posture}}_{i}}∙{\overset{⇀}{a}}^{{\text{Posture}}_{j}}}{∥{\overset{⇀}{a}}^{{\text{Posture}}_{i}}∥∥{\overset{⇀}{a}}^{{\text{Posture}}_{j}}∥} \end{array}$$

We computed coefficient of determination (*R*^2^, Pearson coefficient of correlation, evaluated at significance level α = 0.05) between cosine of the angle between muscle activation pattern vector pairs (Equation 6) and cosine of the angle between corresponding joint torque requirement vector pairs.

#### Effect of generalizability on feasible muscle activation ranges at preferred posture

We tested whether a functional requirement for generalizability across tasks restricts the feasible range of activation in individual muscles to achieve the task at a target posture. We computed the feasible bounds on individual muscles (Sohn et al., 2013) for which a muscle activation pattern satisfied production of experimental synergy force vectors at preferred stance posture while deviations in force directions at test postures were kept within a given tolerance (*Tol*). To examine how restrictions in feasible muscle activation range changes with increasing demand for generalizability, we varied tolerance at three test postures to +10, +5, and +2% of the force angle deviations of the generalizable muscle activation pattern found at preferred stance posture.

The minimum allowable activation for generalizability (${Gen}_{m}^{lb}$) in each muscle was found by solving a non-linear optimization:

(7) $$\begin{array}{rcl} {Gen}_{m}^{lb} & : & Find\;\overset{⇀}{a}\;such\;that\;a_{m}\;is\;minimized,\\ \text{subject to }RF_{AFL}\overset{⇀}{a}=J^{\text{T}}{\overset{⇀}{F}}_{{\text{W}}_{\text{i}}}^{\text{Preferred}},\;{lb}_{m}\leq a_{m}\leq {ub}_{m},\text{ and}\\ △\theta_{{\text{W}}_{\text{i}}}^{\text{Posture}}\leq \text{Tol for shortest},\text{ short},\text{ and long stance}\\ \text{postures} \end{array}$$

Similarly, the maximum allowable activation for generalizability (${Gen}_{m}^{lb}$) in each muscle was found by solving a non-linear optimization:

(8) $$\begin{array}{lll} Gen_{m}^{ub}: & Find\;\overset{⇀}{a}\;such\;that\;a_{m}\;is\;maximized,\; & \;\\ & \text{subject to }RF_{AFL}\overset{⇀}{a}=J^{\text{T}}{\overset{⇀}{F}}_{{\text{W}}_{\text{i}}}^{\text{Preferred}},\;{lb}_{m}\leq a_{m}\leq {ub}_{m},\text{ and } & \;\\ & △\theta_{{\text{W}}_{\text{i}}}^{\text{Posture}}\leq Tol\text{ for shortest},\text{ short},\text{ and long stance } & \;\\ & \text{postures } & \; \end{array}$$

In total, 62 independent optimizations were run (two bounds for each of 31 muscles), resulting in 62 muscle activation patterns for each synergy force vector.

We further tested whether muscles with torque-producing characteristics that were insensitive to changes in posture were preferentially recruited in the generalizable muscle activation pattern. We first computed the sum-squared difference (*SSD*) in torque-producing capability of muscles (i.e., *m*-th column of **RF**_*AFL*_ matrix from Equation 3) between the preferred stance posture and each of the three test postures:

(9) $$\begin{array}{r} {\vec{\text{SSD}}}_{m}=\sum {\left(RF_{AFL}{|}_{m}^{\text{Preferred}}-RF_{AFL}{|}_{m}^{\text{Posture}}\right)}^{2} \end{array}$$

To identify task-relevant changes, we then weighted the differences in torque-producing capacity of muscles at each posture by the sign and magnitude of the required torques for the experimental muscle synergy force vector at the target posture (${\overset{⇀}{\tau }}_{\text{Required}}=J^{\text{T}}{\overset{⇀}{F}}_{{\text{W}}_{\text{i}}}^{\text{Preferred}}$). The task-relevant torque-producing sensitivity was defined by the dot product between the ${\vec{\text{SSD}}}_{m}$ and ${\overset{⇀}{\tau }}_{\text{Required}}$, normalized by the magnitude of each vector, which was identical to the cosine of the angle between the two vectors:

(10) $$\begin{array}{r} {\left[\cos\theta \right]}_{{\vec{\text{SSD}}}_{m},{\vec{\tau }}_{\text{Required}}}=\frac{{\vec{\text{SSD}}}_{m}•{\vec{\tau }}_{\text{Required}}}{\left\|{\vec{\text{SSD}}}_{m}\right\|\left\|{\vec{\tau }}_{\text{Required}}\right\|} \end{array}$$

This weighted each muscle's torque-producing sensitivity based on the relative contributions of the muscle to the joint torque requirements. We examined distribution of torque-producing sensitivity of the muscles and qualitatively compared them with respect to recruitment levels in the generalizable muscle activation pattern.

## Results

In summary, we found that only some of the feasible muscle activation patterns could generalize their force output across postures, demonstrating that generalizability is not a necessary consequence of limb biomechanics. For each case we were able to find a single muscle activation pattern, which we refer to as the generalizable muscle activation pattern, that produced the experimental synergy force vector at target posture and also approximated the direction of the experimental synergy force vectors at the three test postures. The generalizable muscle activation patterns were always suboptimal at each posture in terms of effort, but more generalizable than the min-E muscle activation patterns. Further, we found that generalizability restricts feasible muscle activation ranges of individual muscles, especially for muscles with torque-producing capability that are sensitive to change in postures.

### Generalizability of muscle activation patterns at test postures

**Figure 2:**
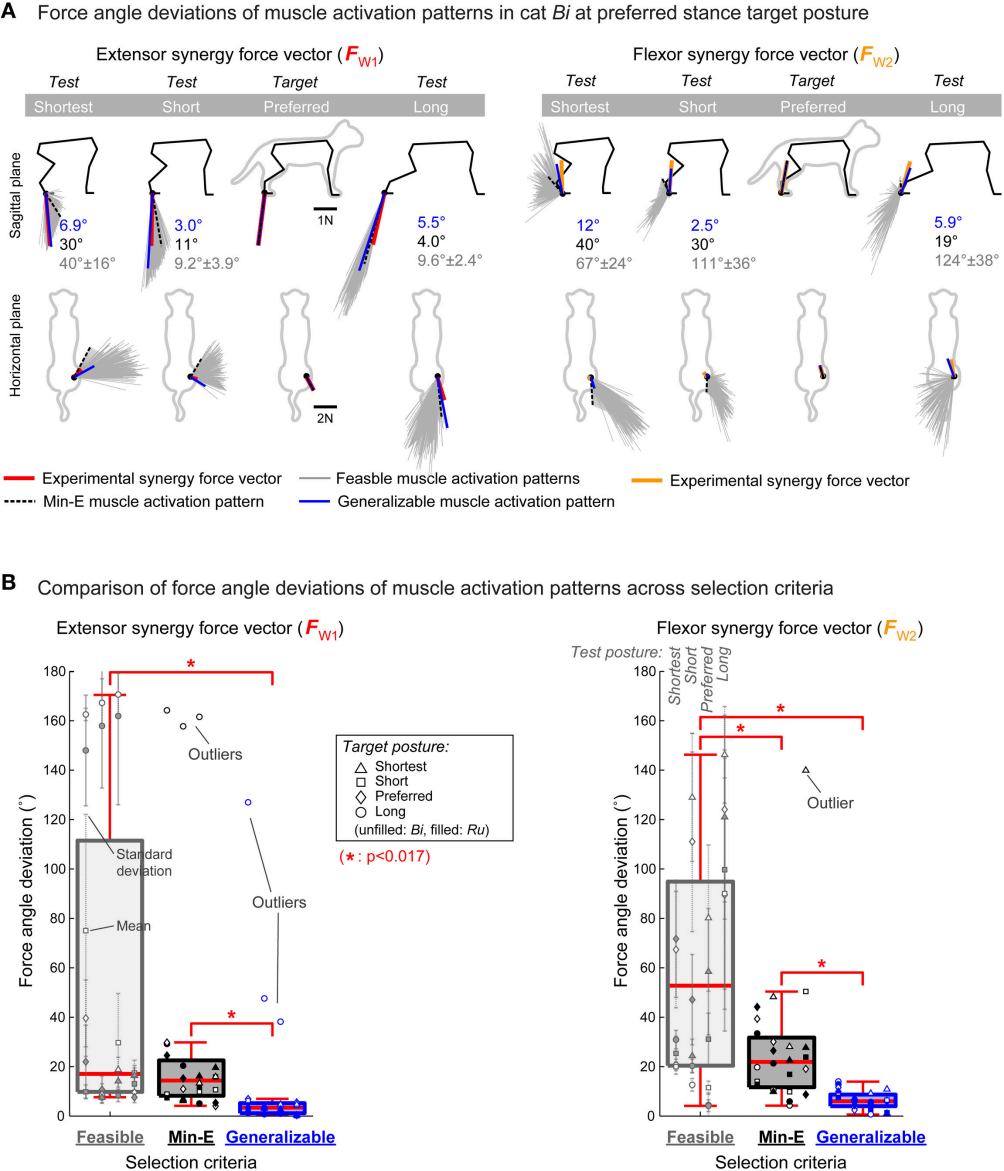
**Generalizability of muscle activation patterns from different selection criteria. (A)** Force angle deviations of muscle activation patterns for the extensor (left, red lines) and flexor (right, yellow lines) synergy force vectors at preferred stance target posture in cat *Bi*. Endpoint force vectors in the test postures are shown with gray lines for the 250 feasible muscle activation patterns, black dotted line for the min-E muscle activation pattern, and blue line for the generalizable muscle activation pattern. The feasible and min-E muscle activation patterns had large force angle deviations, compared to the generalizable muscle activation pattern, and thus could not generalize their function across postures. **(B)** Comparison of force angle deviations of muscle activation patterns from different selection method. Force angle deviations of the generalizable muscle activation patterns were always significantly smaller (^*^p < 0.017) than the feasible and min-E muscle activation patterns across all conditions before and after removing the outliers. Difference between the feasible and min-E muscle activation patterns were not significant for the extensor synergy force vector, and significant for the flexor synergy force vector.

Randomly selected feasible muscle activation patterns did not generalize their function across postures, suggesting that generalizability is not a property of biomechanics of the limb. For example, force angle deviations of the set of 250 feasible muscle activation patterns for the extensor synergy force vector at preferred stance target posture were 40 ± 16° (mean ± std) when tested at shortest stance in cat *Bi* (Figure 2A left, gray force vectors in column “Shortest”). Feasible muscle activation patterns for the flexor synergy force vector at preferred stance target posture were even less likely to be generalizable, especially when tested at long stance: force angle deviations were 124 ± 38° in cat *Bi* (Figure 2A right, column “Long”). Overall, force angle deviations of the feasible muscle activation patterns across all conditions had median of 17° (interquartile range: 9.7–112°) for the extensor synergy force vector (Figure 2B left, gray outline) and 53° (interquartile range: 21–95°) for the flexor synergy force vector (Figure 2B right, gray outline). The force angle deviations of the feasible muscle activation patterns for the extensor synergy force vector at long stance target posture were large (>140°) at all test postures in both cats (Figure 2B left, circles in first column). The feasible muscle activation patterns for the flexor synergy force vector in cat *Bi* also had large force angle deviations (>100°) at shortest stance target posture (Figure 2B right, open triangles in first column) and preferred stance target posture (Figure 2B right, open diamonds in first column) at some test postures. However, there were no outliers for both extensor and flexor synergy force vectors.

The min-E muscle activation patterns were not generalizable across postures in most conditions. For example, in cat *Bi* force angle deviation of the min-E muscle activation pattern for the flexor synergy force vector at preferred stance target posture was 40° when tested at shortest stance (Figure 2A right, black force vector with dotted lines in column “Shortest”). On the other hand, force angle deviation of the min-E muscle activation pattern for the extensor synergy force vector at preferred stance target posture was only 4.0° when tested at long stance (Figure 2A left, black force vector with dotted lines shown in column “Long”). The force angle deviations of the min-E muscle activation patterns across all target postures and cats had median of 14° (interquartile range: 8.1–22°) for the extensor synergy force vector (Figure 2B left, black outline) and 22° (interquartile range: 12–32°) for the flexor synergy force vector (Figure 2B right, black outline). In cat *Bi*, the force angle deviations of the min-E muscle activation patterns for the extensor synergy force vector at long stance target posture were large (>150°) at all test postures (Figure 2B left, open circles in middle column), and were identified as outliers. Similarly, the force angle deviation of the min-E muscle activation pattern for the flexor synergy force vector at shortest stance target posture in cat *Bi* was identified as an outlier when tested at long stance (Figure 2B right, open triangle in middle column).

Force angle deviations of the generalizable muscle activation patterns were generally small (<12°). For example, in cat *Bi* force angle deviation of the generalizable muscle activation pattern for the extensor synergy force vector at preferred stance target posture was less than 3° when tested at short stance (Figure 2A left, blue force vector in column “Short”), and always less than 7° across all test postures. On the other hand, the generalizable muscle activation pattern for the flexor synergy force vector at preferred stance target posture was 12° when tested at shortest stance (Figure 2A, blue force vector in column “Shortest”), which was the largest across all test postures. Across all conditions, force angle deviations of the generalizable muscle activation patterns had median of 3.3° (interquartile range: 1.0–5.1°) for the extensor synergy force vector (Figure 2B left, bar with blue outline) and 6.1° (interquartile range: 4.1–8.8°) for the flexor synergy force vector (Figure 2B right, bar with blue outline). The force angle deviations of the generalizable muscle activation patterns for the extensor synergy force vector at long stance target posture in cat *Bi* was identified as outliers at all test postures (Figure 2B left, open circles in last column).

### Force angle deviation comparison across selection criteria

The force angle deviations of the generalizable muscle activation patterns were smaller than both the feasible and the min-E muscle activation patterns in all of the statistical comparison that we made, including when outliers were removed. Force angle deviations of the generalizable muscle activation patterns were lower than the feasible and the min-E muscle activation patterns (*p* < 0.017) for both extensor and flexor synergy force vectors (Figure 2B). On the other hand, mean force angle deviations of feasible muscle activation patterns were not statistically different from that of the min-E extensor force muscle activation patterns (*p* = 0.14 before and *p* = 0.028 after removing the outliers, respectively; Figure 2B, left). However, the mean force angle deviations of the feasible flexor force muscle activation patterns were larger than the min-E muscle activation patterns (Figure 2B, right).

### Effort level of the generalizable muscle activation patterns

**Figure 3:**
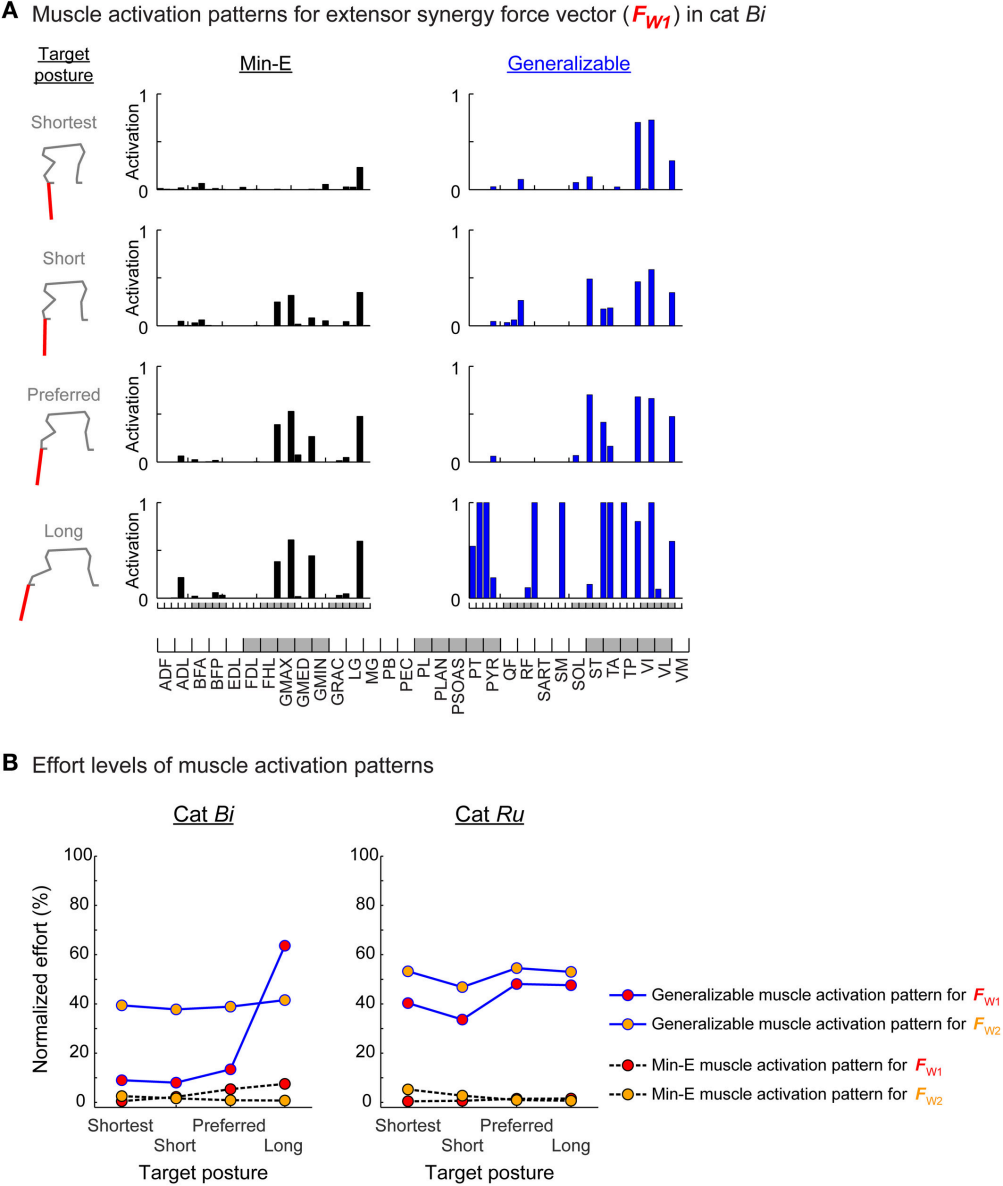
**Muscle patterns and effort levels of the min-E and generalizable muscle activation patterns. (A)** Muscle patterns of the min-E and generalizable muscle activation patterns found for the extensor synergy force vector at each target posture in cat *Bi*. Each bar represents recruitment level of individual muscle in the min-E (left column, black bars) and the generalizable (right column, blue bars) muscle activation patterns. **(B)** Effort level comparisons of the generalizable muscle activation pattern (dots on blue solid lines) and the min-E muscle activation pattern (dots on black dotted lines) at each target posture for the extensor (red circles) and flexor (yellow circles) synergy force vectors. Effort levels were normalized to the maximum possible effort level in each target posture. The generalizable muscle activation patterns were always suboptimal in terms of effort.

The generalizable muscle activation patterns were always suboptimal in terms of effort. The relative effort level of the generalizable muscle activation pattern was greater than the min-E muscle activation pattern in all conditions. For example, in cat *Ru*, effort levels of the generalizable muscle activation patterns for both extensor and flexor synergy force vectors for preferred posture were around 50% of maximum effort, compared to the min-E muscle activation patterns that were less than 5% of maximum effort (Figure 3B right, blue solid lines vs. black dotted lines). Relatively small difference between the effort level of the generalizable and the min-E muscle activation patterns could be found, e.g., for extensor synergy force vector at shortest, short, and preferred stance target postures in cat *Bi* (Figure 3B left, red dots on blue solid lines and black dotted lines). Greater effort of the generalizable muscle activation pattern was due to high activation levels in some of the muscles. In particular, activation levels of more than three muscles were at the physiological maximum activation in the generalizable muscle activation patterns for most conditions (e.g., Figure 3A, last row in right column) except for extensor synergy force vectors in cat *Bi* at shortest, short, and preferred stance target postures (Figure 3A, first to third rows in right column).

### Effect of required torque on similarity in muscle activation patterns across postures

The difference in muscle activation patterns across postures was correlated to differences in joint torque requirements across postures for the min-E muscle activation patterns (*R*^2^ = 0.59 ± 0.27) but not the generalizable muscle activation patterns (*R*^2^ = 0.10 ± 0.09). The highest *R*^2^-value for the min-E muscle activation patterns was found with the flexor synergy force vector in cat *Bi*, which was 0.85 (*p* < 0.05). In contrast, for the generalizable muscle activation pattern for the flexor synergy force vector in cat *Bi, R*^2^ was only 0.12 (*p* = 0.51) between muscle activation pattern and joint torque requirements across postures.

### Effect of generalizability constraint on feasible muscle activation ranges at preferred posture

**Figure 4:**
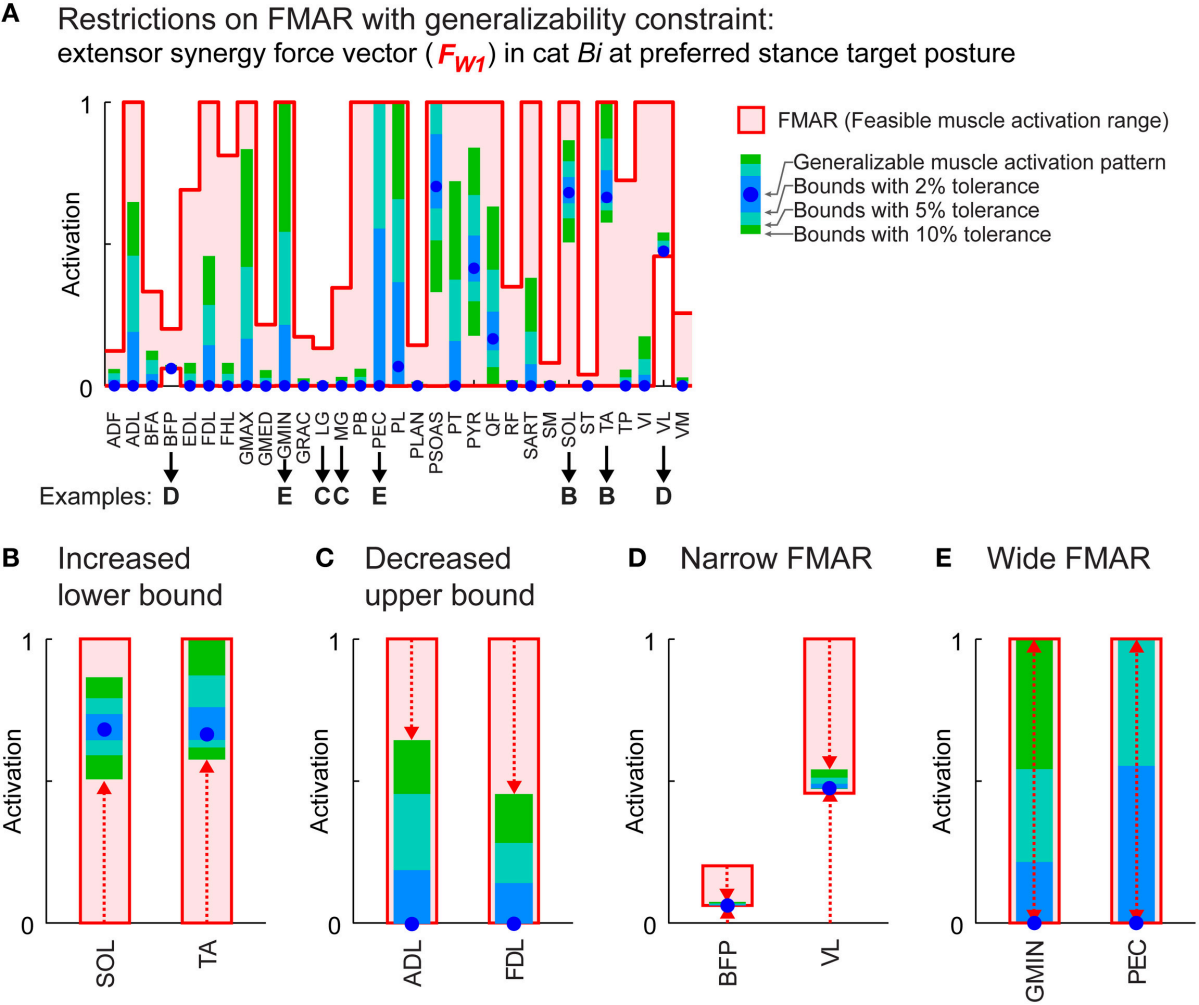
**Feasible muscle activation ranges (FMAR) with generalizability constraint. (A)** Restrictions on the FMAR for the extensor synergy force vector at preferred stance target posture in cat *Bi*. Absolute FMAR is shown as the area between the bold traces that represent the upper and lower bounds. Blue dots show activation level of individual muscles in the generalizable muscle activation pattern. Light blue, blue-green, and green boxes inside the absolute FMAR show the feasible range of activation when force angle deviations at the test postures were allowed to vary by 2, 5, and 10% larger than the generalizable muscle activation pattern. Examples of restrictions on FMAR of the muscles with increased lower bound **(B)**, decreased upper bound **(C)**, narrow FMAR **(D)**, and wide range that were left with great deal of redundancy **(E)**.

The requirement for generalizability across different postures restricted feasible muscle activation ranges. Feasible muscle activation ranges for the extensor and the flexor synergy force vector at preferred posture were reduced by 43 ± 32 and 48 ± 37%, respectively, across all conditions when force angle deviation was allowed to vary by 10% compared to the generalizable muscle activation pattern (Figure 4, green boxes). In general, restrictions were greater with decreased tolerance, e.g., 5% (Figure 4, blue-green boxes) or 2% (Figure 4A, blue boxes), which could happen in several different ways. For example, for the extensor synergy force vector in cat *Bi*, some muscles had an increased lower bound: e.g., SOL and TA (Figure 4B), indicating that these muscles were “necessary” for generalizability. On the other hand, upper bounds were decreased in some muscles: e.g., ADL and FDL (Figure 4C), showing that they were “constrained” with generalizability requirement. Some muscles had very narrow feasible range of activation but nevertheless recruited because of a non-zero lower bound: e.g., BFP and VL (Figure 4D), indicating that they were necessary due to task requirements. Some muscles had wide feasible activation ranges: e.g., GMIN and PEC (Figure 4E), indicating that redundancy remains even with generalizability requirement.

**Figure 5:**
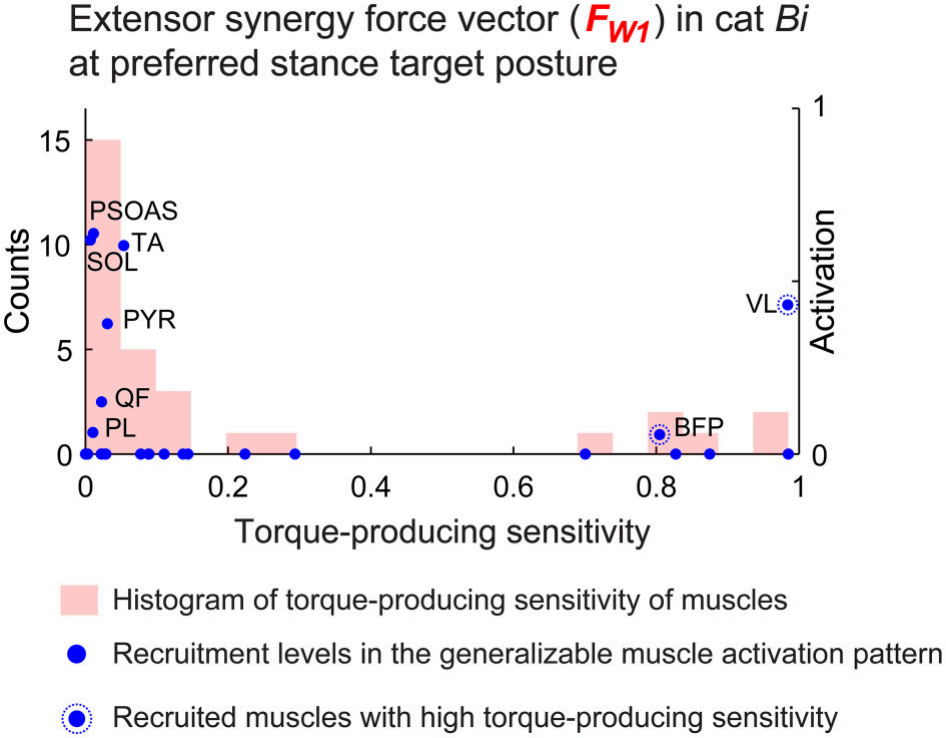
**Torque-producing sensitivity**. Torque producing sensitivity of the muscles (histogram shown with red bars) and recruitment level in the generalizable muscle activation pattern (blue dots) for the extensor synergy force vector at preferred stance target posture in cat *Bi*. Muscles with low torque-producing sensitivity were more likely to be recruited in the generalizable muscle activation pattern, e.g., PSOAS, SOL, and TA.

Muscles with torque-producing characteristics that were less sensitive to change in posture were more likely to be recruited in the generalizable muscle activation patterns (Figure 5, blue dots). Overall, 80 ± 3.1% of the muscles that were recruited (activation>0) in the generalizable muscle activation pattern had relatively low (<0.3) torque-producing sensitivity (Figure 5, X-axis) across all conditions. In particular, muscles that were recruited in the generalizable muscle activation pattern with high activation level had very low torque-producing sensitivity. For example, activation levels of muscles PSOAS, SOL, and TA for the extensor synergy force vector at preferred stance in cat *Bi* (Figure 5, blue dots) were greater than 0.5 and had torque-producing sensitivity lower than 0.1. Some muscles that had high torque-producing sensitivity were recruited in the generalizable muscle activation patterns most likely due to task demands and not the generalizability requirement: e.g., BFP and VL (Figure 5, blue dots circled with dotted lines).

## Discussion

Our results reject two alternative hypotheses about the origin of generalizability of muscle synergies observed experimentally, i.e., ability to use same muscle activation pattern to produce functional motor output across different conditions. First we ruled out the possibility that generalizability of functional muscle synergies arises strictly as a property of the biomechanics of the musculoskeletal system across postures. Our results demonstrate a wide range of possible muscle activation patterns at each posture, many of which do not generalize well in their function when tested across postures. Thus, generalizability is not granted merely by anatomical arrangement and function of limb musculature (Kutch and Valero-Cuevas, 2012). We next ruled out the possibility that generalization of function across postures is a by-product of minimization of effort that render similar muscle synergy patterns across postures. By showing that the optimal muscle activation patterns based on a minimum-effort criteria do not generalize their function across postures we demonstrate that a single optimization criterion (Todorov, 2004) may not be sufficient for the nervous system to organize the spatial structure of muscle synergies. Instead, our analyses suggest that experimentally-identified muscle synergy patterns may arise from explicitly searching for muscle activation patterns that robustly coordinate the limb across postures. This supports our hypothesis based on experimental observations that muscle synergies represent motor patterns selected from among many possible solutions to meet multiple criteria. In contrast to selecting optimal muscle coordination patterns at each condition, selecting more generalizable muscle synergy patterns could reduce the complexity of descending motor control signals necessary for movement.

Our results suggest that muscle synergies may be selected based on generalizability of function produced by muscle activation pattern across multiple conditions. The robustness of muscle synergies that can be generalized across conditions that vary in biomechanical constraints (Hart and Giszter, 2004; Cheung et al., 2005; D'Avella and Bizzi, 2005; Chvatal and Ting, 2013) may support the neural origin hypothesis of muscle synergies, a topic that has been widely debated (Tresch and Jarc, 2009; Hart and Giszter, 2010; Kutch and Valero-Cuevas, 2012; Bizzi and Cheung, 2013). While the latitude the nervous system has in selecting muscle activation pattern for a single sub-maximal task is wide (Martelli et al., 2013, 2015; Sohn et al., 2013; Simpson et al., 2015; Valero-Cuevas et al., 2015), consideration of biomechanical constraints from multiple conditions narrows the range of possible muscle activation patterns that can be generalized across conditions (Loeb, 2000; Keenan et al., 2009; Rácz et al., 2012). We showed that only a few of the redundant muscle activation patterns that satisfy a single task constraint can generalize to other conditions and meet the subsequent task constraints. Tight regulation in force production was required for muscle activation patterns to be generalized in certain conditions, indicating that identifying motor solutions that can be generalized across conditions cannot be guaranteed by satisfying a single task constraint or biomechanical changes corresponding to each task. Thus, muscle synergies may reflect acquired motor solutions that are globally tuned for robustness (e.g., posture-independent) across multiple local conditions, possibly selected from the overlapping region of the solution manifolds from multiple task requirements (Ajemian et al., 2013; Berger et al., 2013; Sadtler et al., 2014).

As biological adaptation processes need to consider robustness and control cost over long time horizons (Clune et al., 2013), it is unlikely that spatial patterns of muscle synergies are organized based on the single optimality principle such as minimizing effort. Rather, muscle synergies may be optimal in a more global sense. They may result from a balance between multiple goals and criteria such as generalizability (Tsianos et al., 2014), computational efficiency or the facilitation of motor learning (Mussa-Ivaldi and Giszter, 1992; Mussa-Ivaldi et al., 1994; Giszter et al., 2007; Berniker et al., 2009; Byadarhaly et al., 2012; McKay and Ting, 2012; Berger et al., 2013). Such solutions need only be “good enough (Loeb, 2012),” rather than optimal, and may have been acquired over an extended period of learning and refinement (McKay et al., 2007; Loeb, 2012; Lacquaniti et al., 2013; Wu et al., 2014). However, muscle synergies may be near optimal with respect to any single criterion. Many modeling studies have shown that spatial organization of muscle synergies resemble muscle activation patterns obtained from optimal control process such as minimizing errors or control effort (Todorov and Jordan, 2002; McKay and Ting, 2012; Steele et al., 2013; De Groote et al., 2014), or that exploits natural limb dynamics (Berniker et al., 2009). By providing predictable input-output behavior, muscles synergies can be flexibly combined and modulated both spatially and temporally according to the task-level goals (Ting and Macpherson, 2005; Lockhart and Ting, 2007; Torres-Oviedo and Ting, 2007; Chvatal and Ting, 2013; Safavynia and Ting, 2013), or across different tasks (Hart and Giszter, 2004; Cheung et al., 2005; D'Avella and Bizzi, 2005). Furthermore, muscle synergies themselves can evolve over time. New muscle synergies can be developed (Dominici et al., 2011; Lacquaniti et al., 2013) or learned (Kargo and Nitz, 2003; Rückert and D'Avella, 2013). Alternatively, spatiotemporal recruitment of pre-existing or acquired muscle synergies can be adapted to novel task requirement or challenges (Cheung et al., 2009; Clark et al., 2010; Berger et al., 2013). Thus, muscle synergies may allow direct control of reliable motor functions that can be used to rapidly adapt motor behavior (Ting and McKay, 2007; Alessandro et al., 2013; Tsianos et al., 2014; Ting et al., 2015).

Wide feasible muscle activation ranges at a given posture provide further evidence that neural selection is involved in shaping muscle synergies. Rather than using musculoskeletal models to search for specific muscle synergy solutions (Kutch and Valero-Cuevas, 2012; Steele et al., 2015), we investigated the set of biomechanically possible muscle activation patterns that could be candidates for muscle synergy solutions as a method to evaluate the role of neural constraints in the appearance of low-dimensional structures in muscle activation patterns. Within the set of muscle activation patterns that were relatively generalizable (e.g., 2~10% larger force angle deviations than that of the generalizable muscle activation pattern), there was sufficient latitude in feasible muscle activation levels that could explain observed individual variations in muscle synergy structure. Further, during searches for a generalizable muscle activation pattern, many local minima were found near the global minimum. For example, for the flexor synergy force vector at long stance in cat *Ru*, there was variability in the activation of muscle PEC (coefficient of variation was 83%) in 77% of the search solutions that started from one hundred initial conditions and converged with cost (i.e., sum-squared force angle deviations across all test postures) not larger than 0.01% of the global minimum. Across all conditions, the variability of solutions near the global minimum depended upon the posture and the animal, with anywhere from 0 to 6 muscles exhibiting coefficient of variation of 12% on average. This redundancy may explain inter-subject variability in muscle synergy patterns that produce essentially same biomechanical output (Torres-Oviedo et al., 2006; Clark et al., 2010; Chvatal and Ting, 2012), as well as deviations of optimal predictions from experimentally-observed muscle patterns (Buchanan and Shreeve, 1996; Thelen and Anderson, 2006). Variability in muscle synergy patterns across individuals may therefore reflect individual differences in habits or preferences (Ganesh et al., 2010; De Rugy et al., 2013), or additional selection criteria regarding energetics (Alexander, 2005; Neptune et al., 2008; Huang and Kuo, 2014) or stability (Bunderson et al., 2008; Liao et al., 2013; Sohn et al., 2013). Our findings may have further implications to more general principles learning motor solutions to novel task by generalizing the function of previously acquired solutions (Tsianos et al., 2014), reflecting the redundant nature of motor control (Loeb and Tsianos, 2015).

In this study, quantitative comparisons of simulated muscle activation patterns to experimentally-derived muscle synergies (Torres-Oviedo et al., 2006) were not possible due to practical reasons. However, the major contributors to each muscle synergy were predicted by simulated muscle activation patterns, i.e., extensors (e.g., VL) for the extensor synergy force vector and flexors (e.g., SART) for the flexor synergy force vector. Further, the minimum-effort solutions exhibited less muscle co-activation than experimental muscle synergies, whereas generalizable muscle activation patterns had more co-activation. However, we note that our minimum-effort solutions for a single force direction appear to have less muscle co-activation than muscle synergies extracted from simulated movement patterns or repertoires (Raasch and Zajac, 1999; Steele et al., 2013; De Groote et al., 2014). But, similar to prior studies, direct comparison of muscle activation patterns are not possible because experimentally-measured muscles are only a small sample of all muscles in the model, and we do not have absolute measure of muscle activation, i.e., the level of EMG associated with maximal contraction force is unknown (McKay and Ting, 2008, 2012; Steele et al., 2013, 2015; De Groote et al., 2014).

We believe that our model-derived estimates of the range of biomechanically-feasible muscle activation patterns that generalize their function across postures is conservative. We used a generic musculoskeletal model (Burkholder and Nichols, 2004) without scaling to animal-specific morphologies, and used common muscle parameters for all conditions. As a result, some muscles in extreme conditions (e.g., long stance) operated at non-physiological ranges and may have caused non-physiological behaviors in our models such as maximal activation in the identified patterns, or large task errors (De Rugy et al., 2013). However, previous studies showed that constraining muscle fiber to more physiological lengths increases muscle feasible activation ranges (Burkholder and Lieber, 2001; Sohn et al., 2013). Further, using subject-specific musculoskeletal models would likely keep muscles within more physiological operating ranges across postures. Adding compliant tendons would also reduce the torque-producing sensitivity of muscles across postures. Other non-linearities such as history-dependence of muscle force generation (Herzog et al., 2000; Hooper and Weaver, 2000; Campbell and Moss, 2002; Siebert et al., 2007) in addition to intrinsic proprioceptive feedback mechanisms have also shown to provide more functional robustness at whole limb level across wider range of biomechanical conditions (Nichols, 1989; Nishikawa et al., 2007; Shemmell et al., 2010). Thus, the set of muscle activation patterns that are generalizable is likely to be wider as the model parameters are refined to be more realistic, allowing for a more continuous and redundant solution space across postures (Zajac, 1989; Wilson and Lichtwark, 2011; Valero-Cuevas et al., 2015).

## Author contributions

All authors listed, have made substantial, direct and intellectual contribution to the work, and approved it for publication.

### Conflict of interest statement

The authors declare that the research was conducted in the absence of any commercial or financial relationships that could be construed as a potential conflict of interest.
